# Comparison of the Lipid Composition of Milk Fat Globules in Goat (*Capra hircus*) Milk during Different Lactations and Human Milk

**DOI:** 10.3390/foods13111618

**Published:** 2024-05-23

**Authors:** Guangqin Liao, Tiancai Wang, Xiabing Li, Jingyi Gu, Qi Jia, Zishuang Wang, Houru Li, Yongzhong Qian, Jing Qiu

**Affiliations:** 1 Key Laboratory of Agri-Food Quality and Safety, Institute of Quality Standard and Testing Technology for Agro-Products, Chinese Academy of Agricultural Sciences, Ministry of Agriculture and Rural Affairs, Beijing 100081, China; lgqlightcheng@163.com (G.L.); 18810583803@163.com (T.W.); lixiabing123452023@163.com (X.L.); msy_361@163.com (J.G.); jiaqi@caas.cn (Q.J.); goldmine_wzs@163.com (Z.W.); qianyongzhong@caas.cn (H.L.); masterhouru@163.com (Y.Q.); 2 College of Food and Biological Engineering, Chengdu University, Chengdu 610065, China

**Keywords:** goat milk, human milk, lactation, milk fat globule, lipid profile

## Abstract

Goat milk is considered the optimal substitute for human milk and is characterized by variations in the lipid composition of its fat globules across lactation phases. Therefore, the objective of this study was to thoroughly analyze the differences between goat milk during different lactations and human milk, aiming to offer scientific guidance for the production of functional dairy products. Compared with transitional and mature milk, the findings indicated that the total membrane protein content in goat colostrum exhibited greater similarity to that found in human milk. Additionally, goat milk exhibited higher milk fat globule size, as well as a higher total lipid and protein content than human milk. A total of 1461 lipid molecules across 61 subclasses were identified in goat milk and human milk. The contents of glycerides and glycerophospholipids were higher in goat colostrum, whereas sphingolipids and fatty acids were more abundant in human milk. Meanwhile, the compositions of lipid subclasses were inconsistent. There were 584 differentially expressed lipids identified between human and goat milk, including 47 subclasses that were primarily involved in the metabolism of glycerophospholipids, sphingolipids, and triglycerides. In summary, for both the membrane protein and the lipid composition, there were differences between the milk of different goat lactations and human milk.

## 1. Introduction

Human milk is widely recognized as a crucial source of nutrition for newborns. Apart from proteins, lipids are the second most important nutrient in raw milk solids, providing 40–60% of a newborn’s energy needs [1]. Furthermore, lipids play a role in the production of bioactive substances that support growth, immunity, gastrointestinal function, and neurodevelopment in infants and young children [1]. However, continuous breastfeeding may not always be feasible because of factors such as illness or an unsuitable work environment. According to the study by Zhao et al., only 35% of infants worldwide are breastfed until six months of age [2]. Hence, it is extremely important to develop functional dairy products that mimic the lipid composition of human milk, such as infant formula (IF), capsules, and tablets [3].

Several studies have indicated that goat milk is considered a highly desirable alternative to human milk and serves as a primary ingredient in functional dairy products [4]. The global market for goat milk is projected to generate approximately USD 15 billion in revenue by 2024, indicating a rising demand for goat milk and its dairy products [5]. The proteins and fats in goat milk are reported to be more digestible compared with cow’s milk produced under similar circumstances [6]. Similarly, goats and sheep have smaller fat globules in milk than cows, which is conducive to milk digestibility and lipid metabolism [7]. Pulina et al. [5] reported in their research that the ratio of milk–meat–wool in dairy sheep is 28:45:27 (38:62 milk–meat, without wool), whereas the milk–meat ratio in dairy goats is 58:42. In China, dairy goat production is increasing rapidly, producing near 2% of the world’s goat milk, and a considerable amount of goat milk is currently used to make milk powder. Therefore, goat milk was chosen as the research object in this study.

The stage of lactation is closely associated with the nutritional composition of milk. Kuchtík et al. [8] identified a decline in polyunsaturated fatty acids during lactation, suggesting that there is a contentious debate surrounding the lipid distribution in goat milk during various lactation periods. Nonetheless, these studies still demonstrate that the stage of lactation significantly impacts lipid distribution. Therefore, to effectively select goat milk at the appropriate lactation period for the production of functional dairy products and IFs, it is imperative to thoroughly investigate the disparities in milk fat globule (MFG) composition between goat and human milk during different lactation periods.

Lipids in human milk and animal milk exist in the form of MFGs, which are primarily composed of triglycerides at their core. The surface of these MFGs is mainly made up of polar lipids and a small amount of glycosylated proteins, which are involved in various physiological responses in the body, forming a complex three-layer membrane structure known as the milk fat globule membrane (MFGM) [9]. The MFGM is about 10–50 nm thick, accounting for up to 6% of MFG mass [9]. Additionally, since IF lacks components like the MFGM present in human milk and undergoes processing that alters the surface composition and particle size of MFGs [10], its efficiency is significantly hampered in terms of lipid digestion and absorption by infants and young children [9]. The secretion and composition of MFGs are usually regulated by the proteins of mammary epithelial cells, such as casein, MFGM protein, etc. Moreover, these proteins also affect the nutrition of functional foods [11,12,13]. As a result, there is increasing interest in MFGs [14,15]. Consequently, a comprehensive understanding of the composition differences in MFGs between goat and human milk is crucial for the development of functional dairy products and IFs.

To the best of our knowledge, there are limited studies available regarding the lipid composition disparities in MFGs between goat milk during different lactations and human milk. Currently, research on the lipid composition of MFGs in goat and human milk primarily focuses on several aspects including the following: investigating the impact of processing methods on the structure and proteins of MFG [16,17], comparing the composition of MFGs between human milk and IF [18], simulating the lipid composition of human milk for the size and distribution of MFGs [19], and examining differences in the lipid composition of human milk fat during different lactations [20,21]. Based on the research background outlined above, our study aimed to thoroughly analyze the differences in lipid compositions of MFGs between goat milk during different lactations and human milk, employing non-targeted lipidomics techniques.

The specific aims of our study were, firstly, to investigate lipidomic characteristics and their differences in goat milk during different lactations thoroughly. Secondly, to evaluate the differences in lipid composition between goat and human milk, which is considered the benchmark for infant nutrition, by comparing the lipid compositions of goat milk at various lactations with those of human milk. Finally, to acquire more information about the composition of milk fat globules in goat and human milk, this research also conducted a comprehensive analysis of the size of MFG and MFGM proteins, providing a scientific basis for the more accurate and effective consumption of goat milk by infants and young children at different times, fully utilizing functional lipids to promote healthy nutritional growth.

## 2. Materials and Methods

### 2.1. Sample Collection

This study was approved by the Ethics Committee of the Chinese Academy of Agricultural Sciences. The classification of lactation periods in goats was based on the reference standard NY/T2835-2015—Technical Specification for Feeding and Management of Dairy Goats [22]. Eight samples of goat colostrum (1–20 days parturition, named Goat-E), eight samples of transitional goat milk (21–120 days parturition, named Goat-M), and eight samples of mature goat milk (121 days parturition to dry milking, named Goat-L) were collected from a small farm in Zibo City, Shandong Province, China. All goats were approximately 2 years old and weighed around 50 kg. They were fed according to a regular schedule in the same enclosure or grazed together in the same pasture, with free access to water and the opportunity to exercise the rest of the time. The formula for feeding the livestock consisted of 20% soybean meal, 40% silage, 20% maize stalks, 10% maize, and 10% wheat bran. Eight samples of human milk (1–7 days postpartum, named HM) were obtained from eight healthy female volunteers aged 25–30 years from a hospital in Zibo City, Shandong Province. These volunteers provided written informed consent indicating that their human milk would be used only for scientific research and confirming that they did not use drugs prior to collection. The number of samples and postpartum days were referenced following the study conducted by Wang et al. and Wu et al. [23,24]. All raw milk samples were shipped within 1 day and stored at −80 °C until experimental analysis.

### 2.2. Reagents and Chemicals

The product information is as follows: acetonitrile (ACN) and isopropanol (IPA) of mass spectrometry grade were acquired from Fisher Scientific (Waltham, MA, USA). Formic acid and ammonium acetate of mass spectrometry grade were purchased from Sigma-Aldrich (Shanghai, China). Dichloromethane, methanol, and hexane of chromatography grade were purchased from Merck (Darmstadt, Germany). Ultrapure water was obtained from a Milli-Q system (Millipore, Billerica, MA, USA). Sodium hydroxide was purchased from Sigma-Aldrich (St. Louis, MO, USA). Lipid internal standard (SPLASH^®^ LIPIDOMIX^®^ Mass Spec Standard 330707-1EA) was purchased from Avanti Polar Lipids (Alabaster, AL, USA).

### 2.3. Protein Measurement

The method for determining membrane proteins in goat milk and human milk followed the protocol described by Zhao et al. [25]. The samples were diluted tenfold with ultrapure water. Subsequently, the diluted samples were mixed with loading buffer (5× containing 2-mercaptoethanol, Mei5bio, Beijing, China) in a volume ratio of 4:1 and then boiled for ten minutes. Afterward, 15 μL of the prepared sample was loaded onto a precast sodium dodecyl sulfate–polyacrylamide gel electrophoresis (SDS-PAGE, Mei5bio, Beijing, China) gel consisting of a polyacrylamide ranging from 4% to 20%. The gel was stained using Coomassie Blue dye (Mei5bio, Beijing, China) and subsequently destained using ultrapure water. Finally, the gel was visualized and analyzed using of an AlphaImager EP system (Santa Clara, CA, USA) and Image J 1.51j8 software (Bethesda, MD, USA).

Protein content was determined using a BCA protein assay kit (Beyotime Biotechnology, Shanghai, China), with all procedures carried out strictly according to the instructions. Each experiment was conducted using an Infinite M200 PRO (TECAN, Männedorf Switzerland) microplate reader [26].

### 2.4. Particle Size and Total Lipid Content Analysis

The particle size distribution was obtained using Mastersizer 2000 equipment (Malvern Instruments Ltd., Malvern, UK). For detailed procedures, please refer to the study by Marie-Caroline Michalski et al. [27]. The particle size was described by volume (D_4,3_).

Total lipids were first extracted using Folch’s method, and the crude fat content of goat milk (without lipid internal standard) at different lactations and human milk was determined following the gravimetric method used by Wu et al. [26]. The formula for the calculation is given below (Equation (1)):

Lipid content (%) = weight of lipid/weight of sample × 100(1)

### 2.5. Lipid Analysis Methods

#### 2.5.1. Lipid Extraction

The lipid extraction method was slightly modified from the Folch method [28]. Briefly, the samples were thawed at 4 °C. Then, 500 µL of the sample and 50 µL of the lipid internal standard were taken, added to the extractant (dichloromethane/methanol, *v*/*v*, 2:1), and vortexed for 1 min. Following ice incubation for 20 min and the addition of 400 µL of water, the mixture was vortexed for 1 min and incubated on ice for another 10 min. The mixture was then centrifuged at 10,000 rpm for 10 min at 4 °C, and the lower layer was transferred into a new centrifuge tube for further analysis. The remaining solution and precipitate underwent a second extraction (without lipid internal standard) following the same procedure. The two lower extracts were combined, dried under nitrogen at room temperature, and finally redissolved in 500 µL of dichloromethane/methanol (*v*/*v*, 1:1). The solution was then passed through a 0.22 µm polytetrafluoroethylene (PTFE) filter membrane for online analysis with UHPLC-Q-TOF-MS.

#### 2.5.2. Lipid Analysis by UHPLC-Q-TOF-MS

Lipid analysis was performed using an ultra-high-performance liquid chromatography (UHPLC) system (Exion LC, AB Sciex, Framingham, MA, USA) coupled with a Triple time-of-flight mass spectrometry (TOF-MS) 6600 (Q-TOF, AB Sciex, Framingham, MA, USA). Lipid extracts were separated by a Phenomen Kinetex 1.7 μm C18 100 Å column (100 × 2.1 mm, Phenomenex, Torrance, CA, USA) in both the positive and negative ionization modes. The mobile phase consisted of ACN:H_2_O (60:40) for A and ACN:IPA (90:10) for B, with both phases including 10 mM ammonium acetate and 0.1% formic acid. The elution gradient was set as follows: 0 min, 40% B, 4 min, 50% B, 25 min, 100% B, 27 min, 100% B, 27.1 min, 40% B, and 30 min, 40% B. The flow rate was 0.3 mL/min. The sample tray temperature was maintained at 15 °C and the column chamber at 45 °C. The injection volumes were 2 µL and 6 µL for the positive and negative ionization modes, respectively. The Triple TOF-MS 6600 mass spectrometer settings were adapted from Li et al. [29].

#### 2.5.3. Lipid Identification and Quantification

For the UHPLC-Q-TOF-MS analysis, a quality control (QC) sample was prepared by mixing 20 μL of lipid extract from each sample. A QC was inserted after every 10 samples to ensure the stability of the instrument. The mass spectral raw data from the samples were processed for peak picking, peak alignment, and ion identification processes using MS DIAL. The software’s parameterization was detailed with reference to Siddabasave Gowda et al. [30], using the following parameters: minimum peak height of 1000 amplitude, mass slice width of 0.1 Da, smoothing level, and minimum peak width of 3 and 5 scans, sigma window value of 0.5, signal intensity 5 times greater than the blank, and MS1 tolerance of 0.015 Da. The annotation of lipids was confirmed by a mass accuracy of less than 5 ppm and percentage matching between experimental and theoretical MS/MS spectra. Based on this, MS2 annotation was conducted using characteristic fragments provided by MS DIAL for each lipid class to eliminate false positive lipids.

Based on the lipid molecules identified by the above methods, we analyzed the fatty acid composition in glycerides, glycerophospholipids, and sphingolipids. We counted the fatty acid (FA) distribution of acyl chains in these three lipid categories in goat and human milk. The number distribution of each type of FA was determined according to the classification method of FA in order to elucidate the distribution of different types of FAs in these three lipid categories in goat and human milk.

Lipid quantification was performed using the peak area and response factor (RF) data (Equation (2)) for each lipid, based on the concentration of the added IS and its peak area, as described by Wang et al. [31]. The RF data are shown in Table S1.

(2) Clipid=CIS ×AlipidAIS×RFlipid

### 2.6. Statistical Analysis

All analyses of lipid subclass contents were performed in triplicate, and the results were presented as the means ± standard errors of the mean (SEM) using GraphPad Prism 8. A one-way analysis of variance (ANOVA) with Duncan’s multiple comparison test was used to compare differences in Goat-E, Goat-M, Goat-L, and HM (* *p* < 0.05, ** *p* < 0.01, *** *p* < 0.001). A principal component analysis (PCA), orthogonal partial least squares discriminant analysis (OPLS-DA), hierarchical clustering analysis (HCA), and pathway analysis of each lipid class and molecular species were performed via MetaboAnalyst 5.0 (https://www.metaboanalyst.ca/) accessed on 10 February 2024. and SIMCA 14 software (Umetrics, Umea, Sweden). Variable importance in projection (VIP) > 1, *p* < 0.05, and fold change (FC) > 1.5 or < 0.67 were used as the reference standards to identify biomarkers. The normalized data were log10-transformed and autoscaled. Cytoscape (version 3.9.1, Bethesda, MD, USA), Microsoft Office Excel 2019 (Microsoft 2019, Redmond, WA, USA), and Origin 2021 (Origin Lab Corporation, Northampton, MA, USA) were used for network diagram and graphics processing.

## 3. Results and Discussion

### 3.1. Interface Protein Composition in Goat Milk from Different Lactation Periods and Human Milk

**Figure 1:**
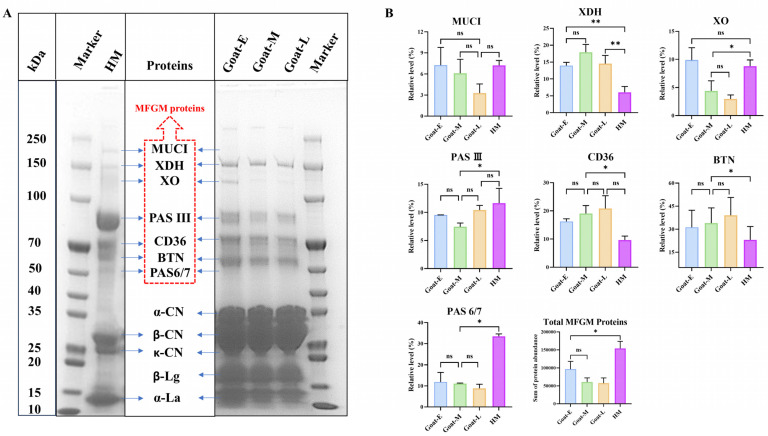
Protein composition (**A**) and level (**B**) in goat milk and human milk. MUC1: mucin 1, XDH: xanthine dehydrogenase, XO: xanthine oxidase, PAS III: high-iodine Schiff glycoprotein III, CD36: platelet glycoprotein 4, BTN: butyrophilin, PAS6/7: periodic acid Schiff 6/7, α-CN: α-casein, β-CN: β-casein, κ-CN: κ-casein, β-Lg: β-lactoglobulin, α-La: α-lactalbumin. ns: not significant. * is *p* < 0.05, ** is *p* < 0.01, “ns” is no significant.

MFGM proteins, which constitute approximately 1–4% of the total protein in milk, are associated with MFG secretion and lipid synthesis, and their content varies across different species [9]. Hence, the difference in MFGM proteins between human and goat milk was determined based on protein gel electrophoresis (Figure 1A). All protein names were referenced according to the research conducted by Fontecha et al. [32].

As illustrated in Figure 1A, the identified membrane protein included mucin 1 (MUC1), xanthine dehydrogenase (XDH), xanthine oxidase (XO), plasma adiponectin surfactant protein III (PAS III), cluster of differentiation 36 (CD36), butyrophilin (BTN), and plasma adiponectin surfactant protein 6/7 (PAS6/7), covering the majority of identified membrane protein species [33,34]. MUC1 prevents pathogenic bacteria from adhering to oral epithelial cells and the colon [35]. XDH is involved in generating reactive oxygen species related to inflammatory responses and antimicrobial activity [36]. XO has antibacterial effects in the intestine [33], and CD36 has been proved to be associated with inhibiting breast cancer [33]. BTN and PAS6/7 are involved in lipid metabolism and transport [37]. Furthermore, the interaction among XDH, XO, and BTN is crucial for the secretion of lipid droplets by mammary epithelial cells [9], promoting the formation of complexes between MFGM proteins and lipids. Therefore, these proteins can participate in the formation of MFG and regulate their particle size.

Figure 1B reveals that XDH, XO, and BTN show varying alterations in goat milk during different lactations and human milk, which may result in different sizes and compositions of MFGs. Because of the inconsistent changes in the relative content of these membrane proteins among the four types of raw milk, a comparative analysis was conducted on the total protein content in the MFGM (Figure 1B). The result showed that the content of membrane protein in human milk was significantly higher than that in goat milk during lactation. Compared with the transitional and mature stages, the membrane protein content of goat colostrum increased significantly. This suggests that certain enzymes in goat mammary glands became inactive during late lactation, leading to a decrease in membrane protein abundance and related lipid synthesis, as corroborated by Thum et al.’s research [9]. According to Pan et al. [34], production and processing would lead to the loss of some membrane proteins in IF. Hence, under identical processing technologies, functional dairy products produced with goat colostrum as a milk source retain more residual membrane protein than those from transitional or mature milk, making goat colostrum the optimal MFGM supplement for IF or other functional dairy products. The same results were also yielded in the study by Huang et al. [38].

Lactoglobulin, casein, and lactalbumin, are the most abundant proteins in milk and play significant roles in the body, partially adsorbing onto the milk fat globule membrane [39]. Figure 1A also demonstrates that casein and lactalbumin were predominant in both human and goat milk, with goat milk exhibiting a higher concentration of lactoglobulin. Moreover, lactalbumin decreased gradually with the increase in the lactation period in goat milk, while casein and lactoglobulin showed no clear change trend. A higher ratio of casein to lactalbumin and β-casein to α-casein is reported to be more beneficial for the rapid digestion of milk proteins in dairy products [40]. In this study, the ratio of casein to lactalbumin and β-casein to α-casein was higher in goat colostrum. This could suggest that the casein-to-whey protein ratio in goat milk presents an appealing alternative for natural components in IF, with even greater benefits observed in colostrum. These results are consistent with those of Fontecha et al. [32], who showed applicable benefits not only to infants but also to other age groups.

### 3.2. Size Distribution in Goat Milk from Different Lactation Periods and Human Milk

**Figure 2:**
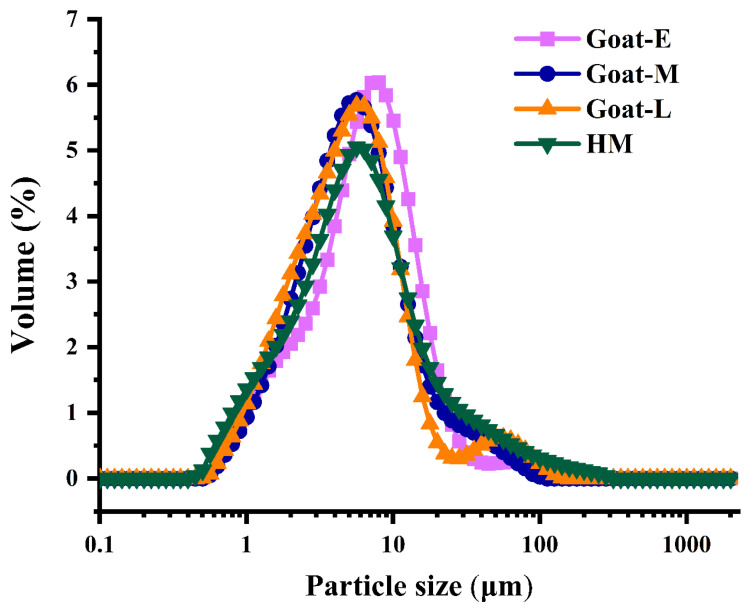
Particle size distribution of MFGs in human milk and goat milk.

Because MFG sizes vary among different species, and the size of MFGs also influences their lipid composition to a certain extent [9]. Therefore, we also conducted measurements of MFG sizes in goat milk during different lactation periods and in human milk, as presented in Table 1 and Figure 2. These size changes are directly correlated with variations in protein and lipid profiles. MFG size can be described by diameter, volume, number, and surface area [9]. The main descriptors include the number-weighted mean (d_n_, D_1,0_), the volume-weighted mean (d_vm_, D_4,3_), and the surface-weighted mean (d_vs_, D_3,2_). In this study, D_4,3_ was chosen to represent MFG size.

The results revealed that HM exhibited the smallest particle size (4.70 μm), while Goat-E displayed the largest particle size (6.03 μm). Goat-M (5.17 μm) and Goat-L (5.31 μm) demonstrated comparable particle sizes, aligning with earlier studies [41]. Similarly, it has been observed that as lactation progresses through different stages of goat milk production, the size of MFGs decreases [9]. The variation in MFG size observed at different stages of lactation may be attributed to the mother’s optimization of maternal energy and the provision of the required nutrients and bioactive components to maximize infant survival [42]. Notably, the Goat-M and Goat-L samples were identified, but all the samples had no statistical difference in particle size, possibly related to the stability of the lipid–protein ratio, indicating that the secretion of proteins and lipids is in equilibrium [9]. This phenomenon was consistent with the relative changes in membrane protein content (Figure 1B). Furthermore, the elevated levels of glycosylated proteins (MUC1, BTN, and XDH) in the MFGM may contribute to the resistance of MFGs against gastrointestinal digestion [9]. Smaller MFGs contain a higher proportion of easily digestible glycosylated proteins than larger MFGs [38]. Although human milk had smaller MFG sizes, its total amount of glycosylated proteins was lower than goat colostrum. Unfortunately, there are currently no clear comparative results on the extent to which MFG size and glycosylated protein content affect MFG digestion.

### 3.3. MFG Lipid Composition in Goat Milk from Different Lactation Periods and Human Milk

#### 3.3.1. Reliability of Analytical Methods

**Figure 3:**
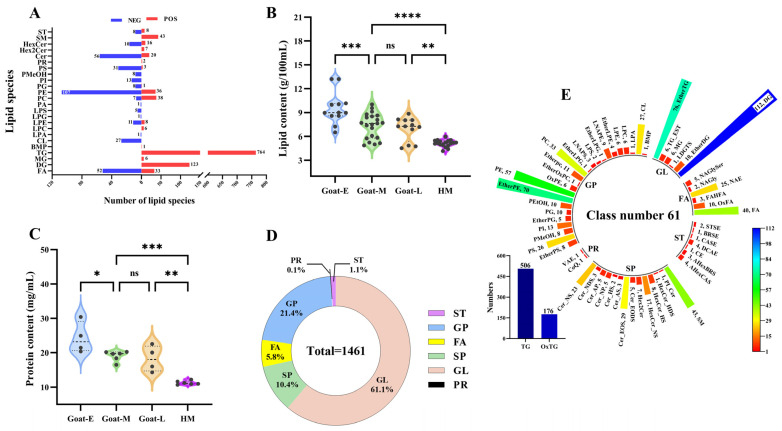
Lipid molecule composition in goat milk and human milk: (**A**) the number of lipid molecules identified in the positive (POS) and negative (NEG) ion modes; (**B**) the lipid content and (**C**) protein content of goat milk and human milk; and (**D**) the number of lipid categories and (**E**) the number of lipid subclasses in goat milk and human milk. Note: for abbreviations, see Supplementary Materials (Table S4—Abbreviations). * is *p* < 0.05, ** is *p* < 0.01, *** is *p* < 0.001, **** is *p* < 0.0001, “ns” is no significant.

In order to evaluate the reliability of the lipid extraction method and the instrumental analytical method, a total of 32 samples were analyzed along with eight identical QC samples. These QC samples consisted of mixtures from the other samples. Figure S1 shows that the total ion chromatograms (TICs) of the QC samples exhibited significant overlap in terms of retention time and peak height, indicating the stability of the instrumental analytical method. The PCA analysis of the QCs and samples (Figure S2) revealed that all samples exhibited RSD ≤ 20%. Additionally, the HCD plots (Figure S3) demonstrated distinct clustering of each sample group, indicating the robustness of the lipid extraction protocol employed in this study. Based on these analytical approaches, a total of 1109 and 352 lipids were identified in the positive and negative modes, respectively (Figure 3A). The total number of identifications in this study far exceeded the findings from previous studies on lipids in goat and human milk [23], thereby significantly enhancing our understanding of the lipid composition in human and goat milk during different lactations.

#### 3.3.2. Total Lipid Levels and Species in Goat Milk and Human Milk

To assess the differences in lipid composition between goat milk and human milk, we first evaluated the content and lipid species.

Total fat content was assessed in goat milk at various lactations and in human milk (Figure 3B). As goat lactation progressed, the lipid content in goat milk exhibited a decreasing trend, with the content in goat colostrum being significantly higher than that in transitional and mature milk. However, there was no significant change between transitional and mature milk. In contrast, the fat content of human milk was significantly lower than goat milk at each stage. Similar changes in lipid content during lactation were observed in the raw milk of other animals [29], yet contrary to the results for human milk during different lactations [24], underscoring the need for a comprehensive analysis of lipids across various lactation phases. The protein components and regulatory mechanisms involved in the lipid secretion pathway in mammary epithelial cells remain unclear [9]. However, studies have noted that both MFG synthesis and secretion involve proteins [13], indicating a close relationship between the protein content in milk and its lipid content. Subsequently, the analysis of total protein content was conducted on four types of raw milk (Figure 3C), revealing a consistent trend with that of crude fat and thus confirming the involvement of membrane proteins. This suggests that the inactivation of proteases in late lactation leads to a reduction in lipid synthesis within the milk.

The lipid profiles of the four types of raw milk were analyzed initially. The lipid species depicted in Figure 3D primarily consisted of six major groups of lipid categories, totaling 1461 lipid molecules with a mass accuracy of less than 5 ppm. There were 1398 lipid molecules identified in human milk and 1418 lipid molecules in goat milk. Among these groups, glycerides (GLs) accounted for 61.1%, sphingolipids (SPs) for 10.4%, fatty acyls (FAs) for 5.8%, glycerophospholipids (GPs) for 21.4%, sterol lipids (STs) for 1.1%, and prenol lipids (PRs) for only 0.1%. FAs, GLs, GPs, and SPs were the predominant lipid categories observed in both the goat and human milk samples, aligning with previous studies on human milk and goat colostrum [23]. Moreover, this study identified a greater number of lipid molecules, which may be attributed to the inclusion of well-established databases such as Lipid Maps, Lipid Blast, and Lipid Bank in our lipid database, enhancing the annotation of a larger number of fragments. Among these lipids (Figure 3E), GLs exhibited the highest number of lipid molecules, with nine subclasses and a total of 893 lipid molecules. Triacylglycerol (TG) emerged as the most abundant subclass, constituting 56.7% of the total GLs. GPs represented the lipid class with the greatest diversity, comprising 24 subclasses (40%) and a total of 319 lipid molecules. Notably, ether-linked phosphatidylethanolamine (Ether-PE) stood out as one of the most prevalent GP subclasses, accounting for 21.9%. SPs consisted of 14 subclasses with a total of 152 lipid molecules, of which sphingomyelin (SM) was the main subclass with 43 subclasses. STs had seven subclasses with a total of 16 lipid molecules, while FAs and PRs had six and two subclasses with 85 and 2 lipid molecules, respectively. These results were in better agreement with the distribution of the number of lipid molecules in other raw milk, such as raw cow’s milk [31], raw goat milk [6], and human milk [43], and complemented findings from other studies.

### 3.4. Difference in Lipids in Goat Milk and Human Milk

#### 3.4.1. Difference in the Number and Content of Lipid Categories

**Figure 4:**
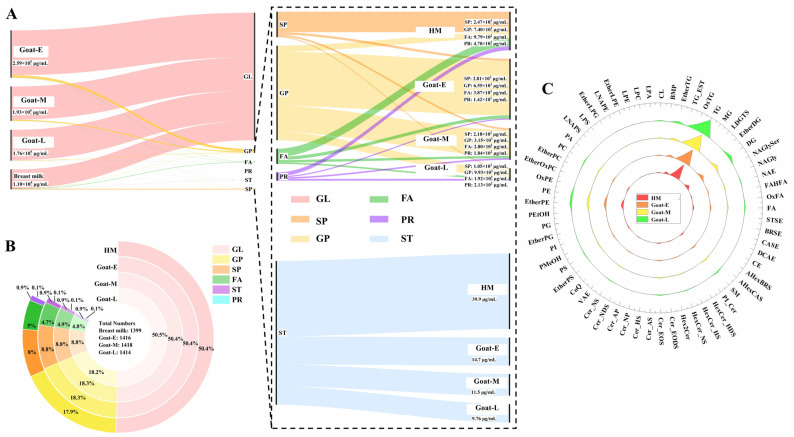
The number and content of lipid categories and classes in goat milk and human milk: (**A**) the content of lipid categories in goat milk and human milk, (**B**) the number of lipid categories in goat milk and human milk, and (**C**) the number of lipid classes in goat milk and human milk.

We further explored the changes in the lipid composition of goat milk during different lactations and human milk. As depicted in the Sankey diagram in Figure 4A, GLs with the largest percentage of content decreased with lactation, with human milk having only 17.5% of goat colostrum’s amount, which was consistent with changes in the crude fat content. The results for other lipids (right side of Figure 4A) showed that SPs tended to decrease across goat lactations but were highest in human milk, reaching up to 2.47 mg/mL, which was 8.8 times higher than colostrum levels. GPs generally decreased throughout lactation, reaching a final level seven times lower than that of colostrum at 0.99 mg/mL for mature milk, yet human milk only reached 74.5% of this level by that time. Interestingly, FAs, PRs, and STs showed a decreasing trend among human milk and different lactations, with STs being the least abundant of these lipids at less than 10%. The amount of each lipid category in each group is identified in the lower right corner of the figures. To our knowledge, this study provides the most comprehensive description yet of the differences between human milk and goat milk regarding their lipid profiles across various stages, including content and categories. Although Zhang et al. [41] studied the surface structure and lipid distribution of goat MFGs during different lactations, only 386 glycerides and 10 phospholipids were identified. Thus, the present study complements their findings.

Although the content of different lipids varied significantly between human and goat milk, there was no significant difference in terms of the number of lipids (Figure 4B). This distribution trend is consistent with the results of other studies on human milk, goat milk, and cow milk [31]. According to Thum’s conclusion [9], goat milk and human milk shared the same MFG structure, but their protein concentration distribution was inconsistent, resulting in different lipid concentrations. The lipid- and protein-related results of this study were consistent with each other. Additionally, goat colostrum, with its higher levels of proteins and lipids, was considered a high-quality dietary supplement. Researchers [9] also indicated that MFGM dietary supplements can improve the functional and adaptive aspects of muscle strength and agility in older adults following exercise interventions. According to the study’s findings, goat colostrum had a higher abundance of MFGM lipids compared with transitional and mature milk, suggesting that goat colostrum could be the optimal MFGM supplement for elderly individuals.

#### 3.4.2. Difference in the Number and Content of Lipid Subclasses

Next, the analysis of the changes in the content of lipid subclasses was conducted. Figure 4C demonstrates the changes in the number of each lipid subclass among different groups, but no significant changes were found. Subsequently, a log 2 transformation analysis was conducted on the content of these subclasses, revealing some interesting phenomena.

**Figure 5:**
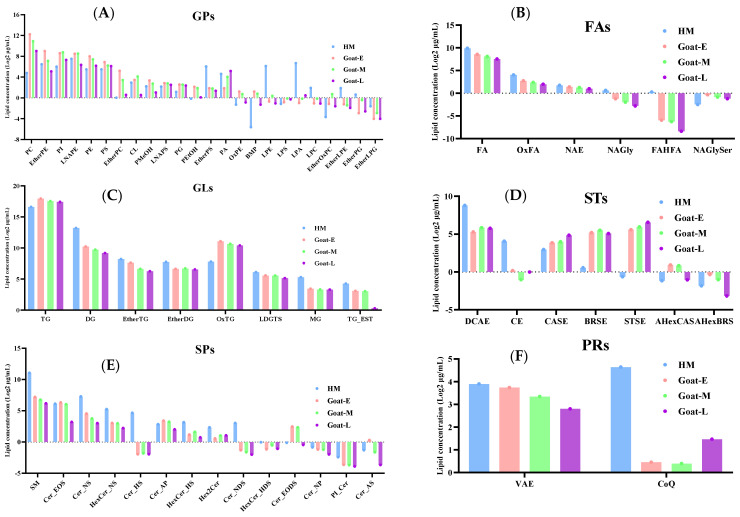
The content of lipid classes for GPs (**A**), FAs (**B**), GLs (**C**), STs (**D**), SPs (**E**), and PRs (**F**) in goat milk and human milk. Note: for abbreviations, see Supplementary Materials (Table S4—Abbreviations).

Among GPs (Figure 5A), 62.5% of the 24 subclasses were at a higher level in goat milk than human milk, except for ether-linked phosphatidylserine (EtherPS), phosphatidic acid (PA), oxidized phosphatidylethanolamine (OxPE), bismonoacylglycerophosphate (BMP), and ether-linked oxidized phosphatidylcholine (EtherOxPC), which all decreased with lactation, while the remaining lipid subclasses were higher in human milk than in goat milk. In addition, phosphatidylcholine (PC) was dominant in GPs, which plays an important role in maintaining the function of biofilms [44]. For babies, the activities of cellular differentiation and division are more active, requiring a large amount of phospholipid synthesis for cell membranes. Therefore, considering the glycerophospholipid content, selecting goat colostrum as the source of polar lipids in infant formula can better fulfill the nutritional requirements for infant growth and development. However, some previous findings showed a dominance of PE, which may be attributed to variability in goat breed and diet [41]. From a physical perspective, PC has stronger surface tension compared with PE. Conversely, PE has a weaker surface. Therefore, the higher the ratio of PC/PE, the greater the lipid droplets and the production of larger particle sizes [45]. According to the results of this study, the ratio of PC/PE gradually decreases during different lactations in goat milk, with the smallest size observed in human milk. Therefore, the trend in particle size changes in human milk and goat milk during different lactation periods was consistent with that of PC/PE lipids, which also confirms the reliability of the previous results (Table 1 and Figure 2).

The changes in FA (Figure 5B) were consistent, with all subclasses being the highest in human milk and showing a decreasing trend with lactation in goat milk, except for N-acyl glycyl serine (NAGlySer). The content of FAs in human milk was consistent with the results of previous studies [46]. NAGlySer are ligands for Toll-like receptor 2 present in the oral bacteria porphyromonas gingivalis, which is related to alveolar bone loss in chronic periodontitis [47].

Regarding GLs (Figure 5C), apart from TG and oxidized triglyceride (OxTG), which were higher in goat milk, the levels of all subclasses followed the same trend as FA in both human and goat milk. Two primary factors contributed to this phenomenon. Firstly, during lactation, monoacylglycerol (MG), and diacylglycerol (DG) undergo significant conversion into other lipids such as TGs and GPs, while also serving as crucial compounds for cell signaling [48]. Consequently, their presence in raw milk was relatively lower. Secondly, this could be related to a decrease in enzyme activity responsible for fatty acid synthesis and the involvement of head- and fat-specific genes in TG storage [46]. In addition, TG serves as a carrier for fat-soluble vitamins such as carotenoids and vitamin E, facilitating the absorption of more nutrients from food by the human body [9]. From this, it could be concluded that goat colostrum has a higher nutritional value.

For STs (Figure 5D), esterified deoxycholic acid (DCAE) emerged as the predominant contributor to the lipid subclass with the highest total ST content in human milk, surpassing goat colostrum by approximately twofold. It has been suggested that elevated cholesterol levels in infants have a long-term effect on cholesterol metabolism, thereby reducing the risk of cardiovascular disease in adulthood [49]. The content of STs in goat milk decreases with the increase in the lactation period, so for infants and young children, using colostrum from goat milk is more beneficial for supplementing ST. However, for patients with atherosclerosis, an increase in cholesterol would worsen their condition [9]. Therefore, fermented milk has been suggested to be more suitable for this group of people.

For SPs (Figure 5E), except for ceramide-esterified omega-hydroxy fatty acid-dihydrosphingosine (Cer-EODS), all lipids exhibited higher concentrations in human milk compared with goat milk and followed the same trend as GLs throughout lactation. Among these lipids, SM was found to be the most abundant in SPs, which is consistent with previous findings indicating its higher abundance in mammals [24]. SM can provide choline, promote the production of neurotransmitters in the brain, and contribute to brain development [50]. Additionally, SM is an important component of cell membranes, forming lipid rafts with cholesterol, which help maintain cell stability and participate in signal transduction [50].

PR levels in human milk (Figure 5F) were consistently higher than in goat milk of any lactation stage, which aligns with the findings of a comparison between human milk and goat colostrum conducted by Wang et al. [23]. PRs have been confirmed as an important structural component of cell membranes and a key signaling molecule in various pathological and physiological states [51].

Overall, the lipid composition of goat milk during different lactation periods differs significantly from that of human milk, which aligns with the findings of Wang et al.’s comparison between human milk and goat colostrum. This difference may be attributed to distinct protein modifications on the endoplasmic reticulum during mammary epithelial cell secretion of MFGs in goats (ruminants) and humans (non-ruminants) [29]. Not only that, but the lipid distribution in goat milk also varies across different lactations, providing a scientific basis for selecting specific lactations in the production of functional dairy products using goat milk as a source.

#### 3.4.3. Differences in the Number and Content of Lipid Molecules

**Figure 6:**
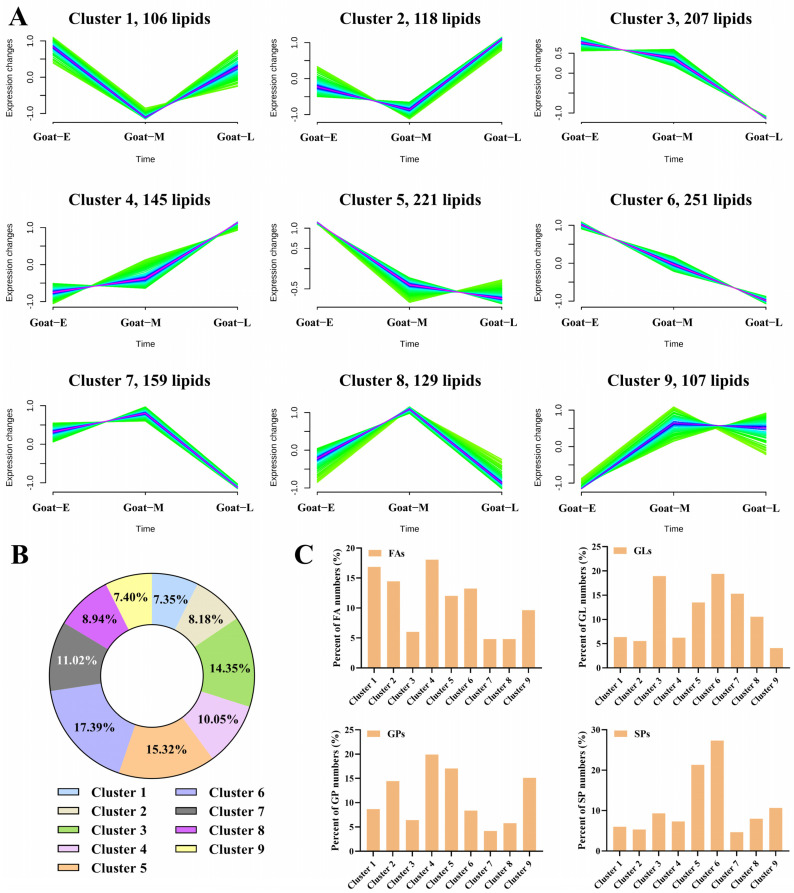
Lipid molecule composition in goat milk: (**A**) k-means cluster analysis of different lipid molecules, (**B**) relative percentages of clusters, and (**C**) relative percentages of FAs, GLs, GPs, and SPs in clusters.

Consequently, k-means cluster analysis was employed to further examine the variation trends in goat milk from different lactation periods for different lipid molecules. Based on species count, these lipids were classified into nine clusters (Figure 6A). Three main trends emerged as follows: first, certain clusters (e.g., clusters 7, 8, and 9) showed an initial increase followed by a decrease with lactation, although the number of lipid molecules within these clusters was relatively small, accounting for only 27.4% of the total lipid count. Second, a decrease followed by an increase in lipid molecule count was observed in other clusters (e.g., clusters 1, 2, and 4), which accounted for 25.6% of the total lipid count. Third, a decreasing trend dominated the overall change pattern in some clusters (e.g., clusters 3, 5, and 6), representing approximately 47.5% of the total number of lipids (Figure 6B). FAs, GLs, GPs, and SPs were the most frequently detected lipid categories (Figure 6C). During lactation, the main trends in FA were rather complex, encompassing the three aforementioned trends (clusters 1, 2, 4, 5, 6, and 9), but the overall trend was decreasing. GPs showed a rising trend (clusters 2, 4, and 9), while GLs (clusters 3, 5, 6, 7) and SPs (clusters 5 and 6) primarily exhibited a decreasing trend. Wu et al. [24] observed a consistent decline in FA levels during human milk lactation, which exhibited a high level of consistency with the decreasing trend seen in goat milk lactation. Consequently, goat milk served as a compensatory source of FAs during the late stages of nursing. Additionally, the trends observed for other lipids were in line with previous findings [41].

### 3.5. Differential Lipids in Goat Milk during Different Lactations and Human Milk

**Figure 7:**
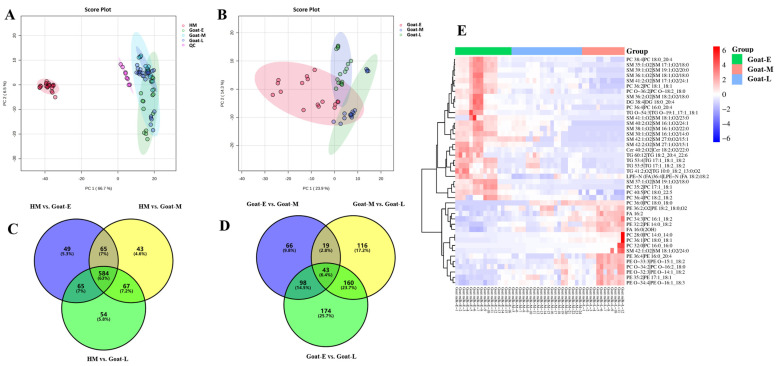
(**A**) Score plot and loading plot of PCA based on goat milk during different lactations and human milk, (**B**) score plot and loading plot of PCA based on goat milk during different lactations, (**C**) content heat map of common differential lipid molecules in the human milk versus goat milk comparison groups and (**D**) the goat milk during different lactations comparison group, and heat map of differential lipid molecules in the (**E**) goat milk during different lactations comparison group.

To thoroughly explore the differences in lipid composition between goat and human milk, we employed multivariate statistical analysis for an in-depth examination. In this study, the PCA model was used to evaluate differentiation based on the mass spectrometry data of each group. As depicted in Figure 7A, a clear distinction between the human milk and goat milk groups was observed, indicating significant variability in emulsion lipids between these two types. The substantial variability in human milk weakened the variation in goat milk lipids across lactations. Consequently, after excluding the human milk samples, a PCA for goat milk from different lactations (Figure 7B) revealed some degree of separation among these groups, suggesting that lactation time impacts emulsion lipid quality. Subsequently, the OPLS-DA discriminant model was employed to identify differentially expressed lipids. To ensure the computational models’ reliability, a permutation test with 200 iterations was performed (Figure S4), demonstrating good fitting and predictive ability for all comparative models.

Differentials between groups were screened based on the following screening criteria: VIP > 1, FC >1.5 or <0.66, and *p* < 0.05. All ion identifications are presented in Supplementary Materials. A subsequent statistical analysis of the differential lipids in all comparison groups of human milk and goat’s milk using Venn plots revealed that 584 lipid molecules were consistently differentiated across all comparison groups (Figure 7C). These lipid molecules encompassed 47 subclasses and belonged to six categories (Supplementary Materials Table S2—HM vs. goat milk). A heatmap analysis (Figure S6) of these 584 differential lipid molecules revealed that 398 lipids (mainly GLs, GPs, and SPs) were up-regulated in each comparison group, while 185 lipids were down-regulated (mainly GLs and GPs), indicating that human milk was less abundant in lipids. Interestingly, in the majority of up-regulated lipids in goat milk, the acyl chains were mainly composed of medium-chain fatty acids (MCFAs) and short-chain fatty acids (SCFAs), while in the up-regulated lipids in human milk, the acyl chains primarily consisted of MCFAs or long-chain fatty acids (LCFAs). This finding aligns with previous studies demonstrating higher concentrations of LCFAs in human milk [23]. In contrast, only 43 common differential lipid molecules were found in the goat milk comparison groups across lactations (Figure 7D). These lipid molecules comprised 14 subclasses and fell into four categories (Supplementary Materials Table S3—Goat milk during lactations). A heatmap analysis (Figure 7E) of these 43 differential lipid molecules revealed that 28 lipids (mainly GPs and SPs) were down-regulated in each comparison group, while 15 lipids were up-regulated (mainly GPs), indicating that early lactation goat milk was more abundant in lipids. This was consistent with the results of fat content and MFG size.

### 3.6. Analysis of Metabolic Pathways

To gain a deeper understanding of the metabolic regulatory pathways involving differentially expressed lipids in goat milk and human milk, we conducted pathway enrichment analysis of the differentially expressed lipids.

**Figure 8:**
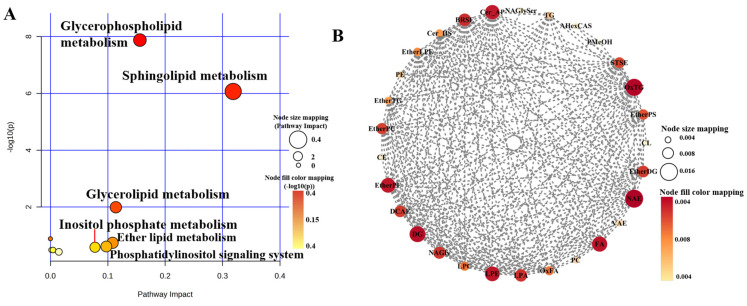

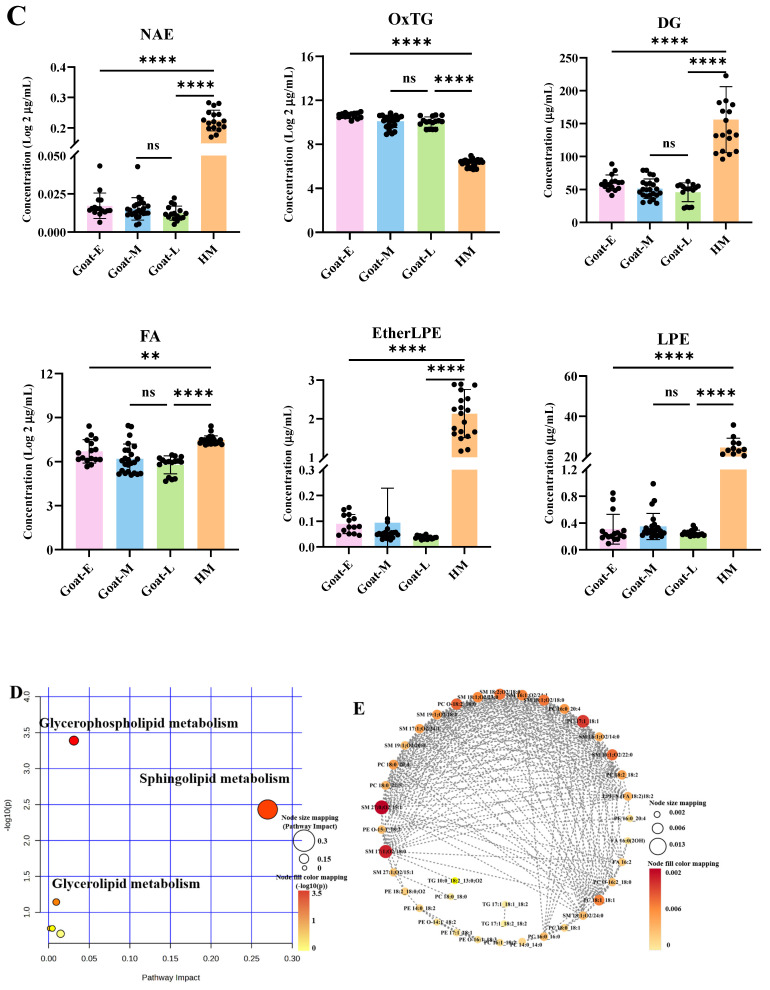

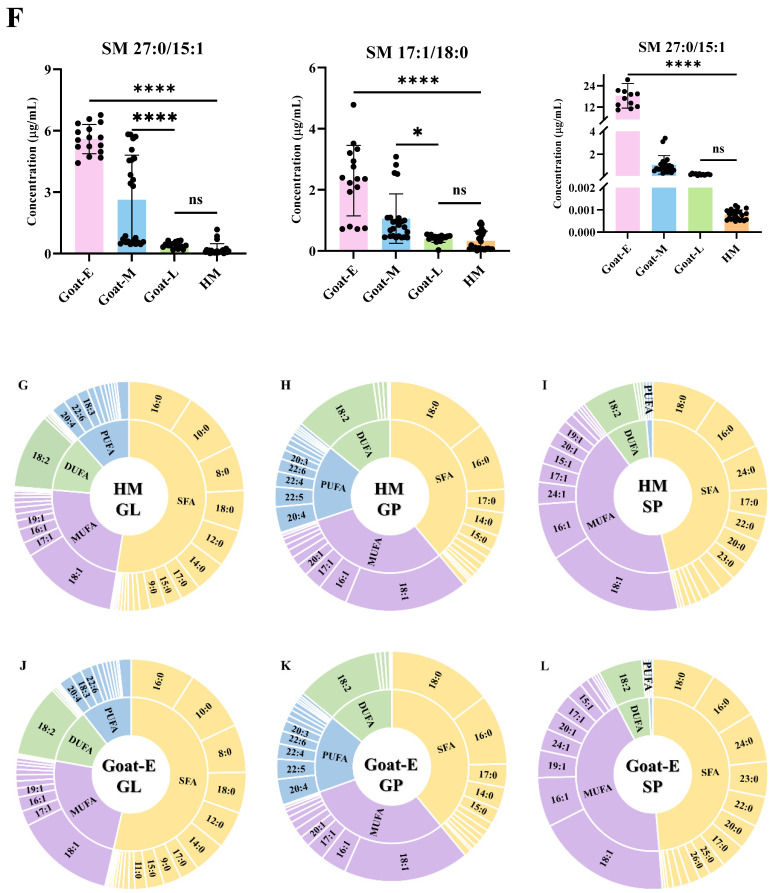
Metabolic pathway analysis of differential lipids. Analysis of metabolic pathways (**A**), molecular correlation network (**B**), and biomarker levels (**C**) of common differentiators in human milk and goat milk comparison groups, analysis of metabolic pathways (**D**), molecular correlation network (**E**), and biomarker levels (**F**) of common differentiators in a comparison group of goat milk during different lactations. Abundance distribution of fatty acids (FAs) in the GL, GP, and SP lipid molecules of HM (**G**–**I**) and Goat-E (**J**–**L**). * is *p* < 0.05, ** is *p* < 0.01, **** is *p* < 0.0001, “ns” is no significant.

Based on significantly distinct lipid metabolites, the lipid molecules were analyzed for their impact on metabolic pathways. As depicted in Figure 8A,D, the differential lipids in both goat milk and human milk influenced the following three metabolic pathways: glycerophospholipid metabolism, sphingolipid metabolism, and glycerolipid metabolism. However, in the comparison between human milk and goat milk, the differential lipids also influenced ether lipid metabolism, the phosphatidylinositol signaling system, and inositol phosphate metabolism. Wang et al. [52] also obtained similar results in their study. Subsequently, a correlation analysis of the differential lipid molecules in each group was performed using MetaboAnalyst 5.0 (http://www.metaboanalyst.ca) accessed on 1 January 2023. (Figure S6). Lipid molecules with correlations greater than 0.9 or less than −0.9 in the comparison between human milk and goat milk were imported into Cytoscape 3.0 for molecular network analysis. Following the methodology of Ma et al. [53], we calculated the scores of each differential lipid using twelve topological analysis methods in CytoHubba and selected those with the highest combined score as biomarkers.

Because of the high number and diverse range of species in the comparison group between human milk and goat milk, the results of differential lipids were aggregated for analysis. Subsequently, it was observed that human milk primarily induced changes in the subclasses n-acylethanolamines (NAE), OxTG, FA, ether-linked lysophosphatidylethanolamine (EtherLPE), and DG compared with goat milk (Figure 8B). Among these lipids, all except OxTG exhibited higher levels in human milk (Figure 8C). NAEs are a crucial class of signaling lipids involved in various physiological processes such as energy homeostasis, anti-inflammatory responses, and neural function [54]. OxTG, naturally present in biological organisms, plays a crucial role in regulating inflammation, cell proliferation, and death programs [55]. Different forms of FA and GP complexes produce different responses in the human brain [56], highlighting the importance of incorporating multiple lipid supplements when modeling human milk composition. In goat milk during lactation, the 43 differential lipid molecules were subjected to network analysis, which identified that SM 27:0_15:1, SM 17:1_18:0, and PC 17:1_18:1 were biomarkers (Figure 8E) as they showed a significant decrease during lactation (Figure 8F). As ruminants primarily feed on grass, goats produce milk fat with higher proportions of odd-numbered acyl chains [57]. The relative proportions of these three sphingolipids in goat colostrum were approximately two and three times higher than in transitional and mature milk, which is consistent with previous results [41]. The high percentage of SM in goat colostrum has been reported to stress indicate its significant potential for promoting early brain myelination formation and neurotransmitter production, as well as possessing anti-cancer, anti-bacterial, and cholesterol-lowering properties [58]. Therefore, goat colostrum could serve as a valuable raw material for the development of health food products aimed at regulating associated functional disorders.

### 3.7. Analysis of Fatty Acid Composition in GLs, GPs, and SPs

The composition of GL, GP, and SP lipid molecules was further studied with higher concentrations in Goat-E and HM, as variations in the fatty acid composition of lipid molecules could lead to variations in dairy product quality and functionality (Figure 8G–L). Given the potential of goat colostrum for IF development, as indicated by previous analyses, this section focuses solely on the FA species analyses of the acyl chains of lipid molecules in HM and Goat-E. The results showed that saturated FAs (SFA), followed by monounsaturated FAs (MUFAs), were predominantly found in the GLs of both human milk and goat colostrum, with similarities in the percentage of contents of di-unsaturated FAs (DUFAs) and polyunsaturated FAs (PUFAs). The FA species distribution in the GLs of the two types of milk was largely similar, yet human milk contained higher levels of docosahexaenoic acid (DHA, C22:6), aligning with the findings of Wu et al. [59]. The most abundant SFAs, MUFAs, DUFAs, and PUFAs in the GLs were palmitic acid (PA, C16:0), oleic acid (OA, C18:1), linoleic acid (LA, C18:2), and arachidonic acid (ARA, C20:4), respectively. The FA distributions of human milk and goat colostrum GPs were also similar overall, but clearly inconsistent with those of GLs, mainly in that the highest SFA abundance was in stearic acid (C18:0). In addition, GPs exhibited a roughly equal abundance of SFAs and MUFAs, which was about double that of PUFAs, and a similar abundance of PUFAs and DUFAs. Given that GPs are a key component of cellular membranes, the unsaturation level of its fatty acids plays a vital role in regulatory and structural functions during infant development, especially regarding long-chain polyunsaturated fatty acids (LCFAs) [60]. Previous studies have shown small quantities of medium-chain fatty acids (MCFAs) and higher levels of LCFAs in human milk [61], which is similar to our results, further suggesting that goat colostrum could be a suitable base for infant and young child formulas in terms of GP composition. The FA profiles of SP in the two types of milk did not significantly differ but were distinctly different from those of GLs and GPs. The proportion of SFAs and MUFAs was about 90%, and the least was PUFAs, suggesting that FAs were less unsaturated in SPs, which may be due to the fact that SPs mainly a signal function, while MUFAs are essential in the development, growth, and maintenance of human health [58].

## 4. Conclusions

In both goat and human milk, seven membrane proteins—MUC1, XDH, XO, PAS III, CD36, BTN, and PAS6/7—were detected. The total MFGM protein content in goat milk was lower than that in human milk, decreasing with prolonged lactation, but the total MFGM protein content in goat colostrum was closer to that in human milk, indicating goat colostrum is a potential optimal source of membrane proteins. Additionally, the MFG size and total lipid content in goat milk during different lactations were higher than those in human milk and decreased with prolonged lactation, indicating notable differences in lipid composition between goat milk during lactations and human milk. Moreover, a total of 1461 lipid molecules, including 61 subclasses, were identified in goat and human milk. The differences in lipid profiles between goat and human milk included 584 lipids and 47 subclasses. Compared with goat milk, human milk had 207 down-regulated and 377 up-regulated lipid molecules. During the different lactations, goat milk exhibited 43 differentially expressed lipids, among which 27 kinds of lipids were up-regulated and 16 kinds were down-regulated in colostrum and mature milk. These differentially expressed lipids were primarily involved in the metabolism of glycerophospholipids, sphingolipids, and triglycerides. Furthermore, analysis of the acyl chains of GLs, GPs, and SPs revealed no significant differences between goat and human milk. Acyl chains with a higher number of SFAs, MUFAs, and PUFAs were found to be highly expressed in GLs, SPs, and GPs, respectively. These findings not only provide reliable data support for the inclusion of goat MFGs in IF but also offer a scientific basis for producing functional dairy products using goat milk.

## Figures and Tables

**Table 1:** Particle size of human milk and goat milk.

Sample	Goat-E	Goat-M	Goat-L	HM
Particle size D_4,3_ (μm)	6.03 ± 0.01 ^a^	5.17 ± 0.33 ^a^	5.31 ± 0.25 ^a^	4.70 ± 0.15 ^a^

^a^ Indicates significant (*p* < 0.05) differences among the samples.

## Data Availability

The original contributions presented in the study are included in the article/Supplementary Materials, further inquiries can be directed to the corresponding author.

## Supplementary Materials

The following supporting information can be downloaded at: https://www.mdpi.com/article/10.3390/foods13111618/s1, Figure S1: Total ion flow chromatograms of human milk and goat milk. Positive mode (A) and Negative mode (B); Figure S2: PCA-X score plots of human milk and goat milk with QCs and other samples; Figure S3: Hierarchical clustering dendrogram (HCD) of human milk and goat milk with QCs and other samples; Figure S4: Parameter evaluation of the orthogonal partial least squares discriminant analysis (OPLS-DA) model by the permutation test (200 times): (A,B) HM vs. Goat-E, (C,D) HM vs. Goat-M, (E,F) HM vs. Goat-L, (G,H) Goat-E vs. Goat-L, (I,J) Goat-E vs. Goat-M, (K,L) Goat-M vs. Goat-L; Figure S5. Heat map of differential lipid molecules in human milk versus the goat milk comparison group. Figure S6: Correlation analysis of different lipid molecules in human milk vs. the goat milk comparison group (A) and the goat milk comparison group during different lactations (B). Red indicates a positive correlation and blue indicates a negative correlation; the darker the color, the greater the correlation; Table S1: The internal standards and calculated RF values used in this study. Table S2. The differential lipids in HM vs. goat milk; Table S3. The differential lipids in goat milk during different lactations; Table S4. Abbreviations.
