# Supplementary material for: Comparison of the Lipid Composition of Milk Fat Globules in Goat (Capra hircus) Milk during Different Lactations and Human Milk
Source: Foods. 2024 May 23;13(11):1618. doi: 10.3390/foods13111618 (PMC11171730; doi:10.3390/foods13111618)
Supplement: Supplementary file 1 [file foods-13-01618-s001.zip › Supplementary material.pdf]

Supplementary Material

Comparative analysis of the differences in fat globule composition between goat (*Capra hircus*) milk at different lactation stages and human milk

Guangqin Liao[1], Tiancai Wang[1], Xiabing Li[1], Jingyi Gu[1], Qi Jia[1] Zishuang Wang[1], Houru Li[1, 2], Yongzhong Qian[1], Jing Qiu[1]*

[1] Institute of Quality Standard and Testing Technology for Agro−Products, Chinese Academy of Agricultural Sciences; Key Laboratory of Agri−food Quality and Safety, Ministry of Agriculture and Rural Affairs, Beijing 100081, China

[2] Chengdu university College of food and biological engineering, Chengdu 610065, China

* Corresponding Author; qiujing@caas.cn

Table S1 The internal standards and calculated RF values used in this study

Lipid class	RF [a]	Polarity
TG+NH_4	1	POS
ST+H	2.33	POS
Sph+H	2.43	POS
SM+H	0.68	POS
PS-H	3.63	NEG
pPE-H	0.89	NEG
pPC+H	0.4	POS
pLPE-H	1.11	NEG
PI-H	1.37	NEG
PhytoSph+H	0.95	POS
PhytoCer+H	1.69	POS
PG-H	1.42	NEG
PE-H	1	NEG
PC+HCOO	0.94	NEG
PA-H	1.57	NEG
LPS-H	1.32	NEG
LPI-H	1.38	NEG
LPG-H	0.8	NEG
LPE-H	0.91	NEG
LPC+H	1	POS
LPA-H	1.68	NEG
HexCer+H	1.53	POS
Hex2Cer+HCOO	2.25	NEG
DG+NH_4	0.88	POS

CL-H	3.21	NEG
Cer-P+H	1.38	POS
Cer+H	2.54	POS
aLPC+HCOO	1.27	NEG

[a] RF is defined as a relationship between the internal standards and an individual lipid class. The RF data are calculated according to the formula below:

$$RF_{lipid} = {Slope_{IS}}\Big/{Slope_{lipid}}$$

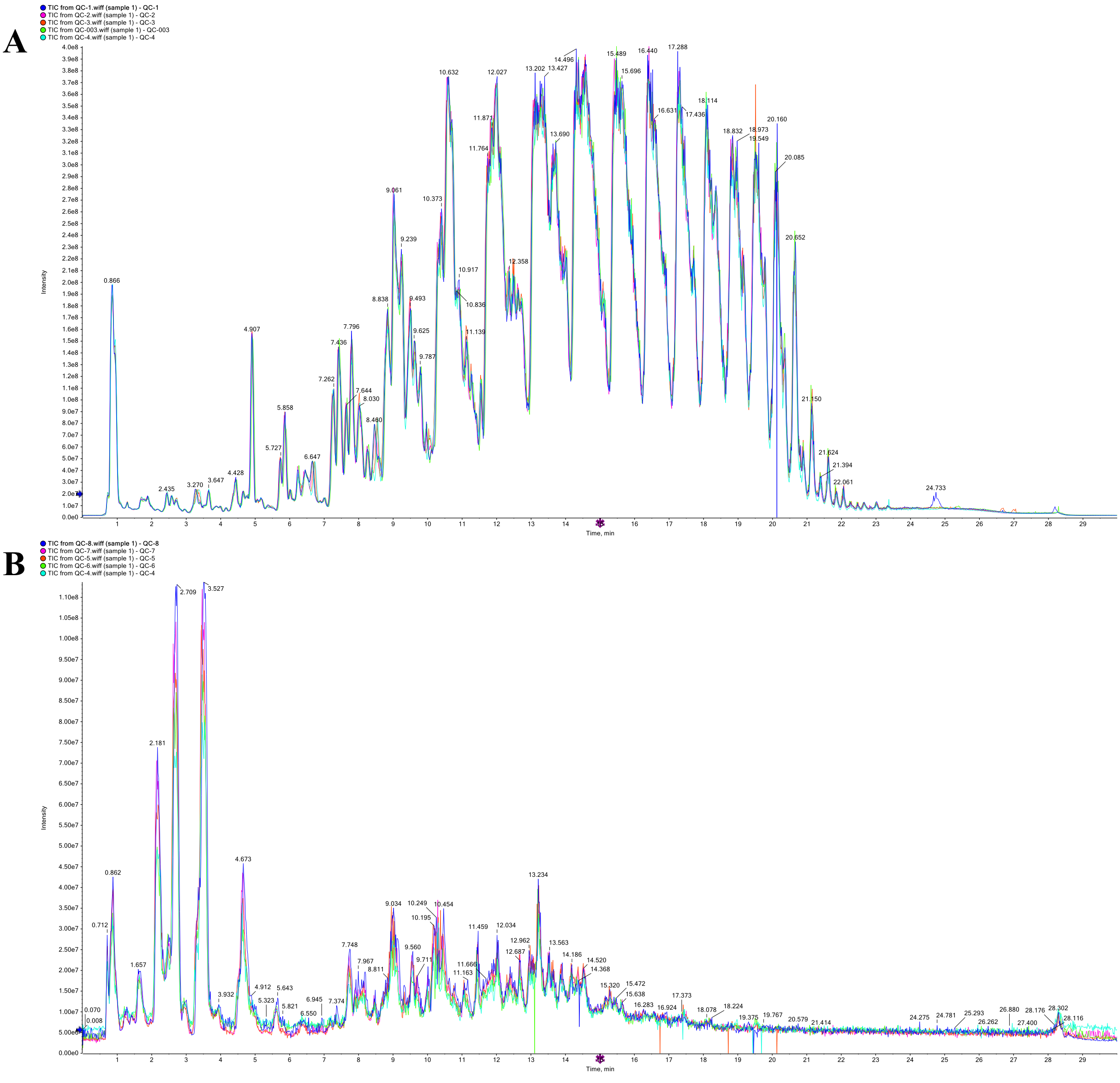

Figure S1. Total ion flow chromatograms of human milk and goat milk. Positive mode (A) and Negative mode (B)

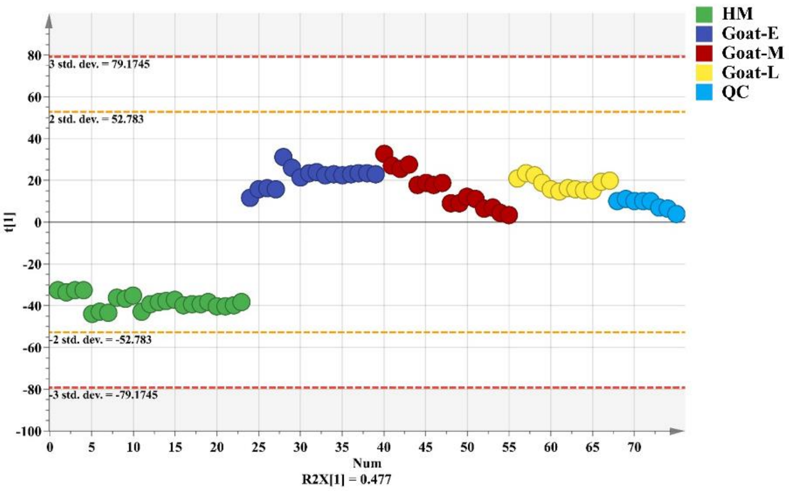

Figure S2. PCA-X score plots of human milk and goat milk with QC and others samples.

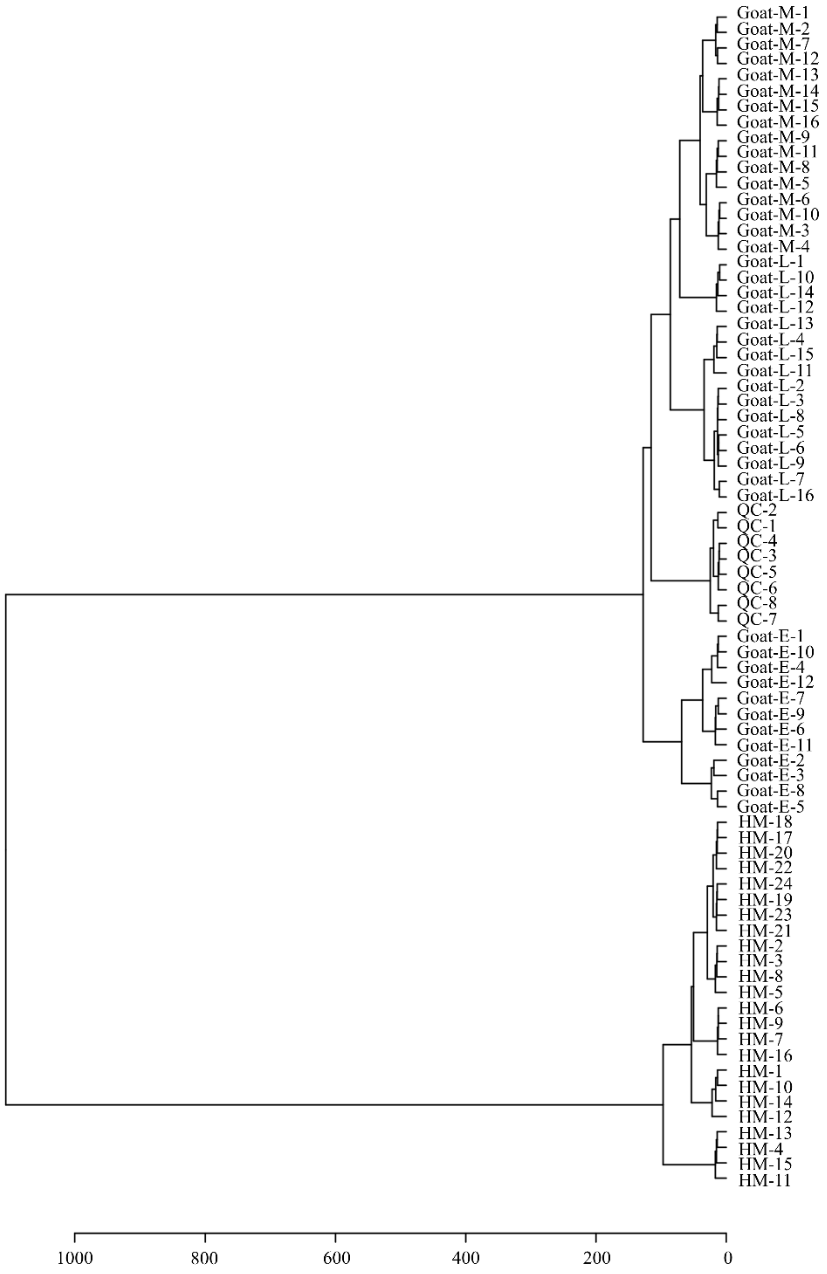

Figure S3. Hierarchical Clustering Dendrogram (HCD) of human milk and goat milk with QC and others samples.

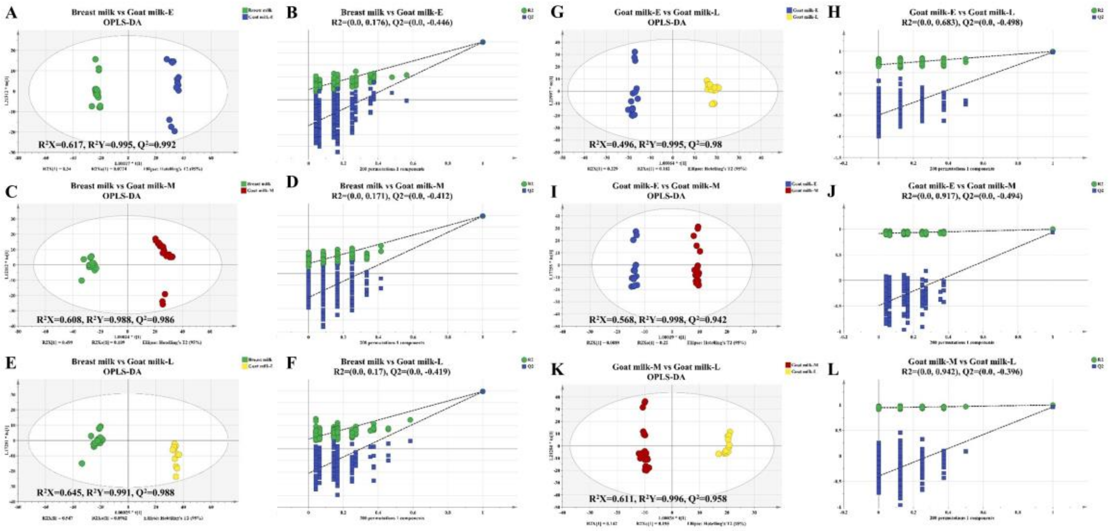

Figure S4. Parameter evaluation of the orthogonal partial least-squares-discriminant analysis (OPLS-DA) model by permutation test (200 times): (A-B) HM vs Goat -E, (C-D) HM vs Goat -M, (E-F) HM vs Goat -L, (G-H) Goat -E vs Goat -L, (I-J) Goat -E vs Goat -M, (K-L) Goat -M vs Goat -L.

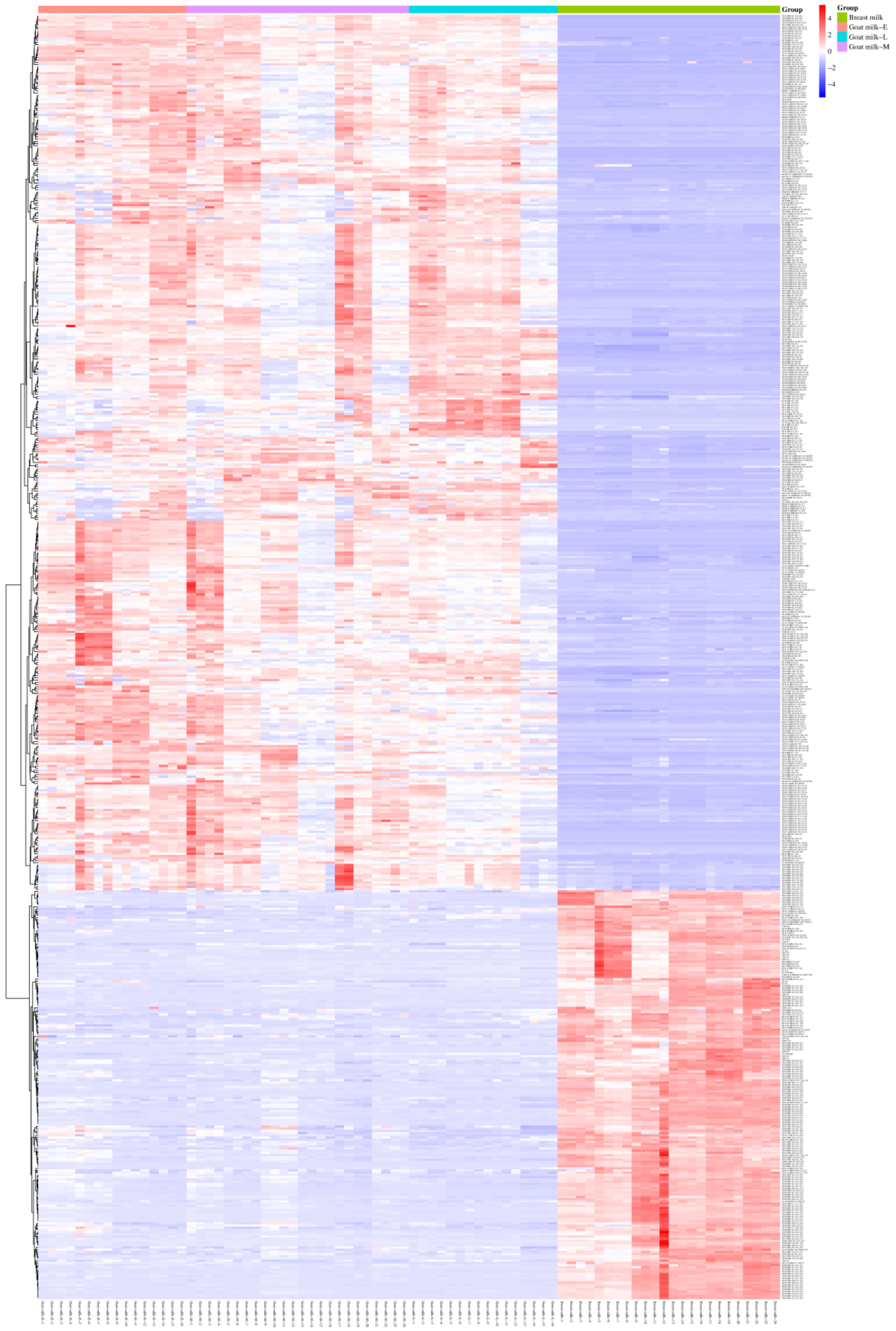

Figure S5. Heat map of differential lipid molecules in human milk versus goat milk comparison group.

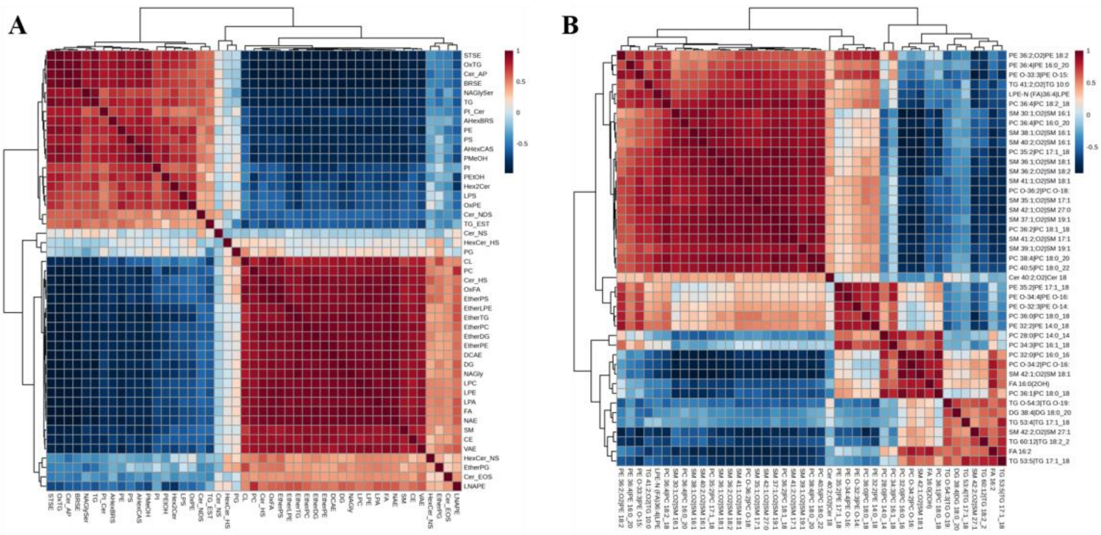

Figure S6. Correlation analysis of different lipid molecules in the human milk vs. goat milk comparison group (A) and the goat milk comparison group during lactations (B). Red indicates positive correlation and blue indicates negative correlation; the darker the color, the greater the correlation.
